# BUDGET IMPACT ANALYSIS OF NONPHARMACOLOGICAL INTERVENTIONS FOR COMMUNITY-DWELLING PEOPLE LIVING WITH DEMENTIA

**DOI:** 10.1093/geroni/igac059.2154

**Published:** 2022-12-20

**Authors:** Eric Jutkowitz, Mauricio Lopez Mendez, Peter Shewmaker

**Affiliations:** Brown University, Providence, Rhode Island, United States; Brown University, Providence, Rhode Island, United States; Brown University, Providence, Rhode Island, United States

## Abstract

Non-pharmacologic dementia care interventions significantly reduce the risk of a nursing home admission for people living with dementia. We used an evidence-based mathematical model to evaluate the budget impact for a healthcare payer that independently implemented four non-pharmacologic dementia care interventions that reduce the risk of transitioning to a nursing home for people living with dementia: 1) MIND, an at-home care coordination intervention; 2) NYU Caregiver (NYUCI), provides caregivers with counseling and ad-hoc support; 3) Alzheimer’s and Dementia Care (ADC) program, a clinic based care coordination intervention; and 4) Adult Day Services Plus (ADS-Plus), an adult day based care coordination intervention. Healthcare payer costs included Medicare and Medicaid expenditures. We simulated a cohort of 302,630 community-dwelling people with dementia, which is the number of people who were diagnosed with dementia in 2018. We applied each intervention’s inclusion criteria to determine the proportion of the cohort that would receive the interventions. Some people may die or enter a nursing home before receiving the interventions. MIND, NYUCI, ADC, and ADS-Plus reduced annual payer expenditures (relative to $25,000, which is the average amount Medicare-Medicaid pay per person with dementia) on average by 0.67%, 0.23%, 0.13%, and 0.58%, respectively over 5 years. Cost savings for the interventions varied by demographics. African American females between ages 95-100 who received NYUCI had the largest cost savings ($2,750.57). White females between ages 65-70 who received ADC had an increase in payer costs ($2,397.07). On average, non-pharmacologic dementia care intervention do not increase a healthcare payer’s budget.

